# Connecting G protein signaling to chemoattractant-mediated cell polarity and cytoskeletal reorganization

**DOI:** 10.1080/21541248.2016.1235390

**Published:** 2016-10-14

**Authors:** Youtao Liu, Jesus Lacal, Richard A. Firtel, Arjan Kortholt

**Affiliations:** a Department of Cell Biochemistry, University of Groningen, Groningen, The Netherlands; b Section of Cell and Developmental Biology, Division of Biological Sciences, University of California, San Diego, La Jolla, CA, USA

**Keywords:** chemotaxis, cytoskeleton rearrangements, *Dictyostelium*, GPCRs, heterotrimeric G proteins, proteomics, Ras, Rap, small G proteins, TORC2

## Abstract

The directional movement toward extracellular chemical gradients, a process called chemotaxis, is an important property of cells. Central to eukaryotic chemotaxis is the molecular mechanism by which chemoattractant-mediated activation of G-protein coupled receptors (GPCRs) induces symmetry breaking in the activated downstream signaling pathways. Studies with mainly *Dictyostelium* and mammalian neutrophils as experimental systems have shown that chemotaxis is mediated by a complex network of signaling pathways. Recently, several labs have used extensive and efficient proteomic approaches to further unravel this dynamic signaling network. Together these studies showed the critical role of the interplay between heterotrimeric G-protein subunits and monomeric G proteins in regulating cytoskeletal rearrangements during chemotaxis. Here we highlight how these proteomic studies have provided greater insight into the mechanisms by which the heterotrimeric G protein cycle is regulated, how heterotrimeric G proteins-induced symmetry breaking is mediated through small G protein signaling, and how symmetry breaking in G protein signaling subsequently induces cytoskeleton rearrangements and cell migration.

Chemotaxis, or directional movement toward extracellular gradient of chemicals, is fundamentally important for processes as diverse as innate immune responses to bacterial infections, finding nutrients, and organizing embryonic structures.^1^ Defects in chemotaxis have been clinically linked to the progression of many diseases including asthma, atherosclerosis, cancer, and several chronic inflammatory diseases. Our understanding of the mechanisms controlling chemotaxis has progressed substantially, mainly through studies targeting specific genes or pathways. Currently there are 2 major viewpoints on chemotaxis; one concentrates on symmetry breaking in intracellular signaling pathways,^2^ while the second concentrates on pseudopods and the physical process that regulates them.^3^ Key to understanding both these viewpoints, which are not mutually exclusive, is to understand how chemoattractants at the outside induce major cytoskeleton changes in the inside of the cell. It is clear that chemotaxis in amoeboid cells, such as neutrophils and *Dictyostelium* cells, starts with binding of the chemoattractant to cell-surface G-protein coupled receptors (GPCRs). The associated heterotrimeric G protein are composed of Gα, Gβ, and Gγ subunits. Upon ligand binding, GPCRs undergo a conformational change that enables activation of the heterotrimeric G protein by GDP to GTP exchange, resulting in the dissociation into Gα-GTP and a Gβγ dimer. This process, in turn, results in the rapid activation of small G proteins, which also switch between inactive GDP-bound and active GTP-bound states. Only in the GTP-bound state can small G proteins interact with downstream effectors. This GDP–GTP cycle is strictly regulated by 2 categories of proteins: guanine nucleotide-exchange factors (GEFs) and GTPase-activating proteins (GAPs).^4^ GEFs facilitate release of the bound nucleotide and allow the more abundant GTP to rebind, whereas GAPs stimulate a small G protein's low intrinsic GTPase activity to stimulate the rate of hydrolysis of the bound GTP to complete the cycle.

In *Dictyostelium*, members of the Ras and Rac family of small G proteins are rapidly and transiently activated at the presumptive leading edge of chemotaxing cells in response to chemoattractant stimulation.^5–9^ In gradients of the chemoattractant cAMP, the receptor occupancy and activation of the receptor-linked heterotrimeric G protein is proportional to the steepness of the gradient,^10,11^ while Ras and Rac activation at the leading edge is much stronger than the steepness of the extracellular gradient.^8,12–17^ These findings suggest that amplification of the extracellular signal and symmetry breaking occurs between heterotrimeric and monomeric G protein signaling. The establishment of an intracellular gradient in monomeric G protein activation leads to major changes in the cytoskeleton: actin polymerization occurs at the leading edge of the cell, while acto-myosin filaments are formed at the rear and side of the cell.^1^ The new actin filaments induce the formation of local pseudopodia, while the acto-myosin filaments inhibit pseudopod formation in the rear and retract the uropod. In addition blebs are formed at the leading edge, probably as a result of the cortical tension forces.^18,19^ Together these cooperative changes in the cytoskeleton result in coordinated cell movement.

The studies so far thus have shown the critical role of the interplay between heterotrimeric G protein subunits and monomeric G proteins in regulating cytoskeletal rearrangements during chemotaxis. But it also raised many new interesting and central questions that must be answered in order to understand directional sensing. How is the heterotrimeric G protein cycle regulated to provide the spatial outputs of Gα and Gβγ? What are the mechanisms by which heterotrimeric G proteins induce activation of monomeric G proteins? What are the connecting components of the core chemotaxis pathway? How is G protein signaling coupled to activation of cytoskeletal elements and subsequently cell movement? We, and others, have adopted comprehensive proteomic approaches to identify additional components of the chemotaxis pathways in order to answer the questions addressed above (Fig. 1).^20–22^

**Figure 1.**
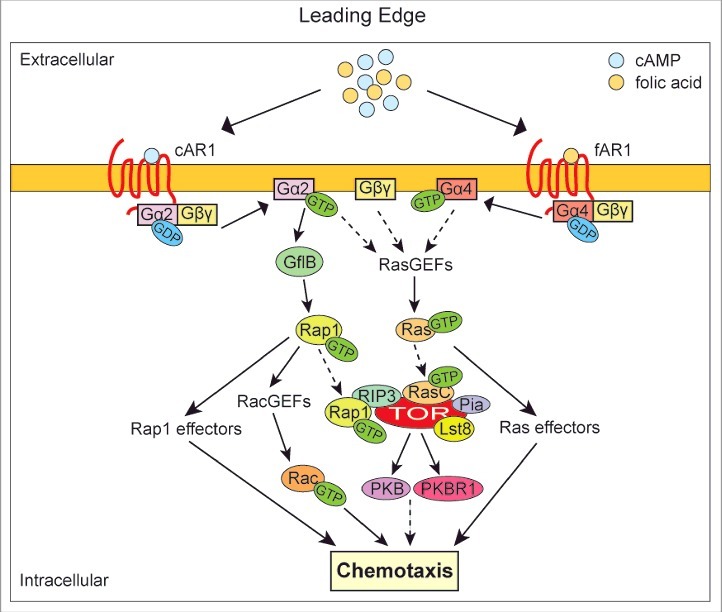
Cartoon depicting the recently identified GPCR-mediated pathways that regulate *Dictyostelium* chemotaxis. In *Dictyostelium* chemotaxis is initiated by binding of the chemoattractants to GPCRs, cAR1 (cAMP receptor) and fAR1 (folate receptor), leading to the dissociation of heterotrimeric G protein into Gα2-GTP/Gα4-GTP and a Gβγ dimer. Subsequently, Gα2-GTP, Gα4-GTP and Gβγ all can regulate Ras signaling via Ras specific GEFs. Moreover, Gα2-GTP can directly interact with its effector GflB to activate Rap1, thereby initiating a subset of downstream singling pathways. TORC2 is a common effector of Rap1 and Ras signaling. RasC directly binds the kinase domain of TOR and Rap1 positively regulates the RasC-mediated activation of TORC2 by binding to RIP3, providing a possible mechanism by which TORC2 integrates the Ras and Rap1 pathways during chemotaxis.

In both mammalian neutrophils and *Dictyostelium*, chemotaxis is initiated by the binding of chemoattractants to cell surface G protein coupled receptors (GPCRs) (Fig. 1). *Dictyostelium* depends on chemotaxis toward folate for chasing bacteria as food source, whereas chemotaxis to cAMP is essential for the development into fruiting bodies upon starvation.^23^ The cAMP receptor, cAR1, was the first chemoattractant GPCR discovered in eukaryotic cells.^24^ In contrast, the folate receptor remained unknown for more than 4 decades after folate was identified as chemoattractant.^25^ Since ligand binding to almost all GPCRs induces the phosphorylation of its C-terminus, Pan et al., generated phosphoproteomic data in the presence and absence of folate to identify the folic acid receptors, fAR1 and fAR2.^22^ Importantly, fAR1 not only controls chemotaxis toward folic acid secreted by the bacteria but it is also essential for phagocytosis of the bacteria. This mechanism may well be conserved as neutrophils might use a similar chemoattractant-mediated engulfment mechanism for the clearance of bacterial infections.

Binding of chemoattractants to cell surface GPCRs results in the rapid GDP-GTP exchange and subsequent dissociation of Gα-GTP and the Gβγ dimer (Fig. 1). From a classical point of view, Gα subunits might be considered to serve as “timer” to govern Gβγ signaling by releasing and re-associating Gβγ dimer from/to GPCRs through GDP/GTP exchange and the subsequent hydrolysis of GTP. As a result, less attention has been paid to direct signaling by the Gα subunit. However, recently, it has been realized that Gα plays an equally important role in transducing signal from GPCRs to downstream effectors as, more and more, Gα-specific effectors in chemotaxis have been identified. For instance, in mammalian neutrophils, Gα_i_ can interact with Elmo1/Dock180,^26^ mInsc,^27^ and Homer3,^28^ while Gα_12/13_ is able to bind to p115RhoGEF^29^ and mTORC2.^30^ In *Dictyostelium*, disruption of Gα2, the Gα subunit that interacts with the cAMP receptor cAR1, results in cells that do not respond to stimulation by the chemoattractant cAMP and are unable to aggregate.^31^ Despite the essential function of Gα2 in cAMP-mediated chemotaxis, Gα2 had not been reported to directly activate downstream chemoattractant effectors in *Dictyostelium*. We identified GflB as the first Gα2 effector in a proteomic screen in *Dictyostelium* by using purified Gα2 protein as a bait.^20,32^ GflB is a Gα2-stimulated Rap1 specific GEF that is required for efficient directional sensing and cell movement during chemotaxis.^16^ Therefore, GflB forms a direct connection between heterotrimeric G protein and monomeric G protein signaling (Fig. 1). GflB binds specifically to Gα2 (cAMP GPCR) and not Gα4 (folate GPCR): the activation of GflB thus provides a mechanism for *Dictyostelium* cells to respond differently to distinct chemoattractants. During chemotaxis to cAMP, GflB accumulates at the leading edge via an actin dependent positive feedback loop mechanism. Translocation of GflB to the cell membrane is initiated by Gα mediated lipid binding of the N-terminal domain of GflB, followed by localization to the cell cortex via binding of the C-terminal domain of GflB. At the leading edge, GflB regulates the balance between Ras and Rap1 activation, which regulates cAMP-mediated cytoskeletal rearrangements, resulting in recruitment of additional GflB to the cortex. GflB thus provides a direct link from Gα activation to localized monomeric G protein signaling and localized cytoskeletal rearrangement. Although human Rap1 was initially identified as a suppressor of Ras signaling, it is now clear that in both mammals and *Dictyostelium* Ras and Rap1 activation are strongly interconnected.^33,34^ Using a proteomic approach, we recently identified the Target of Rapamycin Complex 2 (TORC2) as integrator of *Dictyostelium* Ras and Rap1 signaling in response to chemoattractants (Fig. 1).^21^ TORC2 has conserved roles in regulating cytoskeleton dynamics during chemotaxis in eukaryotes. The *Dictyostelium* TORC2 complex consists of Lst8 (mLst8 in mammals), Rip3 (mSin1), Pia (RICTOR) and Tor (mTor). We found that both Rap1 and RasC activate the TORC2 complex by binding to the RIP3/SIN1, and the catalytic domain of TOR, respectively. The interactions between *Dictyostelium* TORC2 and Ras/Rap1 appear to be conserved in human.^21^ Recent data also suggest that in mammalian cells the TORC2 complex is not only regulated by monomeric G proteins, but also by heterotrimeric G proteins.^35^ These new studies suggest that the highly conserved TORC2 functions to integrate G protein signals to coordinate cellular migrations in many systems. Future studies need to be directed at determining whether the interacting proteins are all activators of the complex or whether some function as scaffold to localize the complex. Is simultaneous interaction with multiple components required for activation of the TORC2 complex (coincidental detector), or can each activator stimulate the enzyme by itself?

The work discussed here has provided important new insights in the molecular mechanisms underlying the regulation and connection of G protein signaling and cytoskeleton during chemotaxis. Interestingly, a recent study revealed that *Dictyostelium* Ras also plays a central role in micropinocytosis, suggesting that these 2 important signaling pathways overlap.^36^ Together this also demonstrates that the use of *Dictyostelium* as model system, in combination with mass spectrometry based proteomic, provides an excellent strategy to get new insights in the molecular mechanisms underlying regulation of intracellular signaling. The observed similarities to pathways in mammalian cells suggest that these mechanisms are highly conserved through evolution and thus presumably apply to normal cell functionality and human disease processes.
